# Comparative Analysis of two Sugarcane Ancestors *Saccharum officinarum* and *S. spontaneum* based on Complete Chloroplast Genome Sequences and Photosynthetic Ability in Cold Stress

**DOI:** 10.3390/ijms20153828

**Published:** 2019-08-05

**Authors:** Fu Xu, Lilian He, Shiwu Gao, Yachun Su, Fusheng Li, Liping Xu

**Affiliations:** 1 College of Agronomy and Biotechnology, Yunnan Agricultural University, Kunming 650201, China; 2 Key Laboratory of Sugarcane Biology and Genetic Breeding, Fujian Agriculture and Forestry University, Fuzhou 350002, China

**Keywords:** sugarcane ancestors, chloroplast genome, genome comparative analysis, cold stress, chlorophyll relative content, chlorophyll fluorescence

## Abstract

Polyploid *Saccharum* with complex genomes hindered the progress of sugarcane improvement, while their chloroplast genomes are much smaller and simpler. Chloroplast (cp), the vital organelle, is the site of plant photosynthesis, which also evolves other functions, such as tolerance to environmental stresses. In this study, the cp genome of two sugarcane ancestors *Saccharum officinarum* and *S. spontaneum* were sequenced, and genome comparative analysis between these two species was carried out, together with the photosynthetic ability. The length is 141,187 bp for *S. officinarum* and that is 7 bp longer than *S. spontaneum*, with the same GC content (38.44%) and annotated gene number (134), 13 with introns among them. There is a typical tetrad structure, including LSC, SSC, IRb and IRa. Of them, LSC and IRa/IRb are 18 bp longer and 6 bp shorter than those in *S. spontaneum* (83,047 bp and 22,795 bp), respectively, while the size of SSC is same (12,544 bp). Five genes exhibit contraction and expansion at the IR junctions, but only one gene *ndhF* with 29 bp expansion at the border of IRb/SSC. Nucleotide diversity (Pi) based on sliding window analysis showed that the single copy and noncoding regions were more divergent than IR- and coding regions, and the variant hotspots *trnG-trnM*, *psbM-petN*, *trnR-rps14*, *ndhC-trnV* and *petA-psbJ* in the LSC and *trnL-ccsA* in the SSC regions were detected, and *petA-psbJ* with the highest divergent value of 0.01500. Genetic distances of 65 protein genes vary from 0.00000 to 0.00288 between two species, and the selective pressure on them indicated that only *petB* was subjected to positive selection, while more genes including *rpoC2*, *rps3*, *ccsA*, *ndhA*, *ndhA*, *psbI*, *atpH* and *psaC* were subjected to purifying or very strong purifying selection. There are larger number of codons in *S. spontaneum* than that in *S. officinarum*, while both species have obvious codon preference and the codons with highest-(AUG) and lowest frequency (AUA) are same. Whilst, the most abundant amino acid is leucine in both *S. officinarum* and *S. spontaneum*, with number of 2175 (10.88% of total) and 2228 (10.90% of total) codons, respectively, and the lowest number is cysteine, with only 221 (1.105%) and 224 (1.096%), respectively. Protein collinearity analysis showed the high collinearity though several divergences were present in cp genomes, and identification of simple sequence repeats (SSRs) were included in this study. In addition, in order to compare cold tolerance and explore the expanding function of this environmental stress, the chlorophyll relative content (SPAD) and chlorophyll fluorescence Fv/Fm were measured. The significantly higher SPAD were observed in *S. spontaneum* than those in *S. officinarum,* no matter what the control conditions, exposure to low temperature or during recovery, and so was for Fv/Fm under exposure to low temperature, together with higher level of SPAD in *S. spontaneum* in each measurement. Aforementioned results suggest much stronger photosynthetic ability and cold tolerance in *S. spontaneum*. Our findings build a foundation to investigate the biological mechanism of two sugarcane ancestor chloroplasts and retrieve reliable molecular resources for phylogenetic and evolutionary studies, and will be conducive to genetic improvement of photosynthetic ability and cold resistance in modern sugarcane.

## 1. Introduction

Chloroplast, the vital organelle, is the site of plant photosynthesis. Generally, for most photosynthetic organisms, chloroplast (cp) genomes range from 115 kb to 165 kb in size [1,2,3]. The cp genomes are conventionally described as a circular DNA, consisting of a quadripartite structure of one large single-copy (LSC) region, two inverted repeat (IRa and IRb) regions, and one small single-copy region (SSC) [4]. Their sequence, organization, gene content and gene order are considered to be well conservative due to an absence of genetic recombination [5,6,7], though there are mutation events, insertions and deletion, pseudogenes or structural gene rearrangements occurrence in some lineages [2,8,9,10,11], the mutation rate is low. Additionally, substitution and base insertion or deletion are the main mutation types. Hence, the cp genome sequence has been used in phylogenetic and evolutionary analysis, identification of SSRs and SNPs, and development of plant barcoding [12,13,14,15,16,17], and defining species limits [18]. 

Sugarcane, which has been cultivated in more than 100 countries, is the most important sugar crop accounting for more than 78% of the total world sugar production. The ‘noble’ species *Saccharum officinarum* and the wild *S. spontaneum* are valuable resources which contributes respectively sugar, and biotic and abiotic stress resistance, and growth vigor. These two species belong to the genus *Saccharum*, which contains six different species including *S. officinarum*, *S. spontaneum*, *S. sinense*, *S. barberi*, *S. robustum* and *S. edule*. Among them, *S. officinarum* and *S. spontaneum* are thought to be the ancestors of modern cultivated sugarcane [19], of which 70–80% from *S. officinarum*, 10–20% from *S. spontaneum* and about 10% from interspecific recombination [20]. Thus, the genomic structure of modern sugarcane hybrids is recognized to have homologous interspecific and intraspecific chromosomes [21], while *S. sinense* and *S. barberi* are regarded as interspecific hybrids between *S. officinarum* and *S. spontaneum* [19], which resulting in an exceedingly complex interspecific polyploid sugarcane genome. Due to the complexity in ploidy, the complete genome of modern sugarcane is still remaining to be deciphered though great progress has been made by drawing allele-defined genome of tetraploid *S. spontaneum* AP85-441 [22], and by assembling a mosaic monoploid reference sequence for modern sugarcane cultivar R570 based on bacterial artificial chromosome (BAC) clones [23]. One common characteristic is their multiple chromosome numbers in the different species and even in the same species of the genus *Saccharum*, which normally is thought to be 2n = 80 for original accessions of *S. officinarum*, in spite of different opinions referring to *S. officinarum* can be found, which including 2n =70 to 140 for *S. officinarum* [24] and the controversial basic chromosome number (× = 8 or 10) [25,26]. Both the homogenous polyploid nature and the large variation of chromosome number in the genus *Saccharum* and in the specie *S. spontaneum*, whose chromosome number ranges from 40 to 128 and ploidy from 4× to 12× [20,24], have increased its difficulty in genome assembling and gene annotation, together with the analysis of their phylogeny. The complex genome of modern sugarcane hinders the progress of its improvement, while their chloroplast genomes are much smaller and relatively simpler, thus, study on sugarcane genome can aid to promote sugarcane improvement, especially in molecular marker selection. 

The first complete sugarcane chloroplast genome was obtained from modern hybrid NCo310 by CEQ Dye Terminator Cycle Sequencing [27], and soon after that, the cp genome of sugarcane hybrid SP80-3280 by shotgun sequencing was obtained [28]. Both carried out a comparative analysis with monocot wheat, rice, and maize chloroplast genomes by focusing on the genes at the *rps16–trnQ* and the *trnS–trnC* regions, respectively, to identify the divergences and polymorphisms. Recently, the complete cp genome of sugarcane hybrid Q155 [6] and RB867515 [29] were obtained, and their main focuses are on identification of polymorphic sites, insertion and deletion events by comparing Q155 with NCo310 and SP80-3280 [6], and RB867515 with Q155, NCo310 and SP80-3280 [29], respectively. All of these studies focused on sugarcane hybrids. More recently, focusing on sugarcane origins from the plastid view was reported by comparing their complete cp genomes of *S. spontaneum*, *S. officinarum* and *Miscanthus floridulus* [30]. However, an understanding of the structure variation, identification of highly divergent regions, analysis of coding protein genes and single nucleotide polymorphic loci in cp genomes between the crucial species, i.e., two ancestor species *S. officinarum* and *S. spontaneum* of modern sugarcane cultivars, is very limited, and needs to be elucidated in more detail.

Plants perform photosynthesis in the chloroplast, which is known as the vital organelle, to acquire energy for growth and development. Whilst, in addition to this crucial function, it also evolves to extend functions, such as tolerance to environmental stresses of drought, temperature extreme, high salinity and alkali, high light strength, ultraviolet radiation and heavy metals [2,31,32,33,34,35,36]. Recently, chloroplast acting as a sensor of environmental signals and playing a key role in the construction of plant immunity were suggested by Serrano et al. [37]. However, the response of sugarcane chloroplasts to environmental stresses is remained to be revealed. Temperature is a critical factor which profoundly affects plant growth, development, and geographical distribution [38,39,40]. Sugarcane originates from tropical regions and is currently mainly cultivated in subtropical regions, such as Queensland in Australia, San Paulo in Brazil, Guangxi in China, after long-term domestication. However, it usually suffers from a serious effect on yield and quality due to its weak cold tolerance when exposed to low temperature [41], and frequency changes of climate in recent years aggravate the effects of low temperature on sugarcane industry. Fortunately, the sensitivity to cold varies widely among sugarcane germplasms [42]. 

*S. officinarum* and *S. spontaneum* in *Saccharum* are two ancestor species of modern sugarcane cultivars or hybrids. Two accessions of Badila and Yunnan 83-184 are the clones belonging to *S. officinarum* and *S. spontaneum*, respectively. Among them, Badila is also used as a cultivar for chewing cane (not for making sugar) although it is an original or primitive cultivar. In this study, we (1) obtained the complete chloroplast genome sequences of *S. officinarum* and *S. spontaneum*; (2) carried out the comparative cp genomics between the ‘noble’ species *S. officinarum* and wild species *S. spontaneum*; (3) elucidated their structure variation, identified the highly divergent regions, compared coding protein genes and estimated their genetic distances, and identified single nucleotide polymorphism (SNP) loci. Additionally, the photosynthetic parameters of SPAD (chlorophyll relative content) and Fv/Fm (variable chloroplast fluorescence/maximal chloroplast fluorescence) in control (before exposed to stress), low temperature and during recovering in field conditions were investigated.

## 2. Results

### 2.1. Quality and Quantity of Raw Data and Clean Data Obtained from Sequencing

Using the Illumina HiSeq 2500 system, 5,701,229,700 and 5,343,719,700 base-pairs of clean data were generated from *S. officinarum* and *S. spontaneum*, respectively, and their reads reach 519× and 316× coverage over the two cp genomes, respectively. Among them, 19,004,099 and 17,812,399 paired-end reads after filtering artificial sequences and low-quality reads (≤Q20 bases) were obtained in *S. officinarum* and *S. spontaneum*, respectively. The high quality of raw data is indicated by the 92.83% and 93.03% of ≥Q30 bases in clean reads in *S. officinarum* and *S. spontaneum*, respectively. Before assembling, chloroplast DNA (cpDNA) sequences were drawn by aligning with the database of plant cp genomes. Only those matched the cp sequences in this database can be used for genome assembling, which are 262,149 reads for *S. officinarum* and 174,306 reads for *S. spontaneum*. The histograms of insert sizes for both species were shown in Figure S1, and the average insert size is 303.7 bp ± 69.4 bp for *S. officinarum*, and 310.6 bp ± 70.7 bp for *S. spontaneum*. The ambiguous nucleotide sites in the cp genome, produced in the scaffold extension step during de novo assembly, were corrected manually. The errors relevant to heterogeneous Indels (i.e., insertions/deletions) caused from homopolymeric repeats in the genome were also corrected. To test the quality of assembly of the cp genomes of *S. officinarum* and *S. spontaneum*, three steps including: (1) using assembled genome as reference, statistical genome coverage, insert fragment size, etc.; (2) genome alignment with the reference sequence (NCBI accession: LS975131.1) to check conservation and rearrangement; (3) alignment with the reference for structure information. The final sequences of complete cp genomes for *S. officinarum* and *S. spontaneum* will be deposited in GenBank, and their corresponding accession numbers are MN204507 and MN204508, respectively, and they will be released on January 26, 2020.

### 2.2. Chloroplast Genomic Structure and Gene Diversification

**Figure 1:**
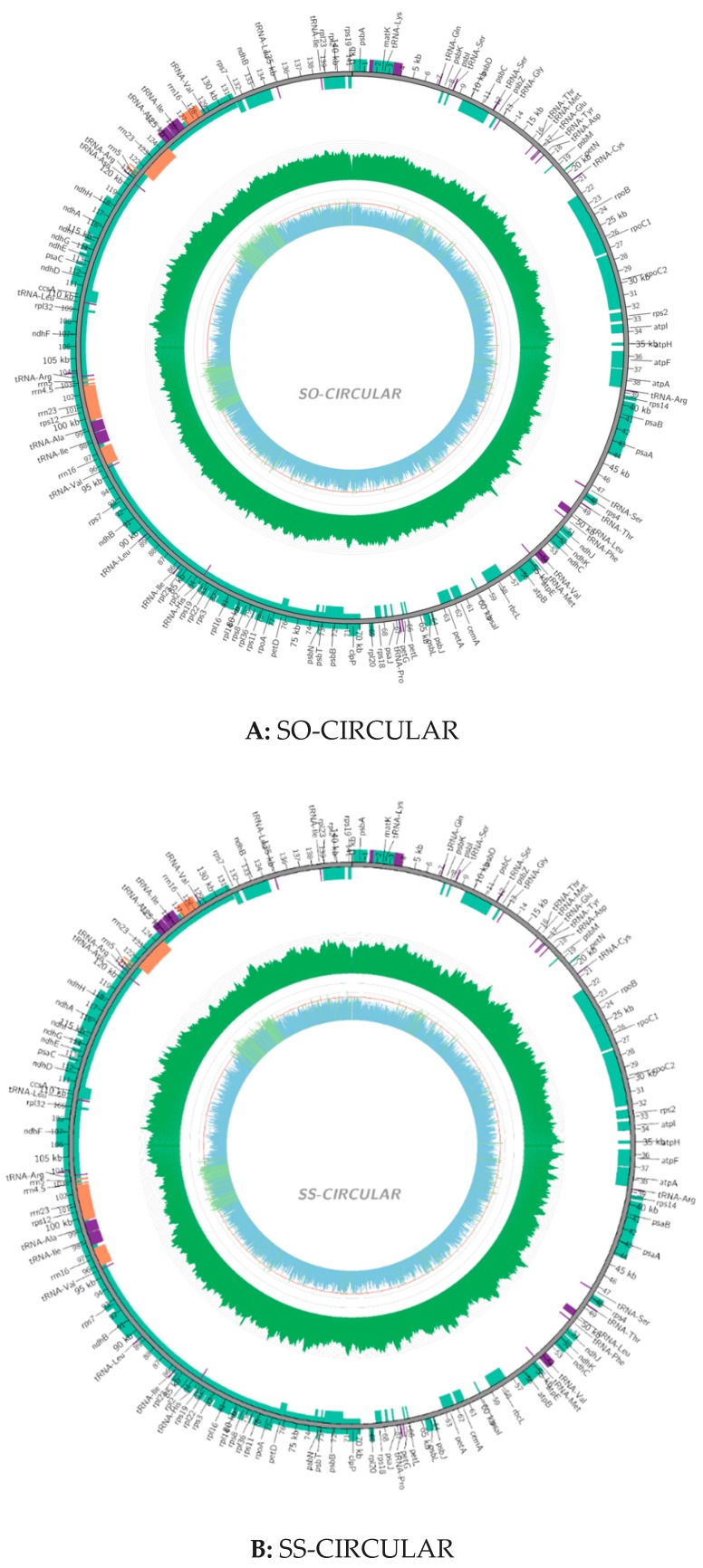
Coverage map for assembly chloroplast sequence genomes of *Saccharum officinarum* (**A**) and *S. spontaneum* (**B**). Outermost circle: genome sequence. Green box: the coding genes; Purple box: tRNA; Orange box: rRNA; Green ring: the coverage depth, of which reverse repeat area is generally two times than that of other areas; Inner circle: GC content of the genome, of which green line representing greater than 50%, and blue for smaller than 50%.

**Figure 2:**
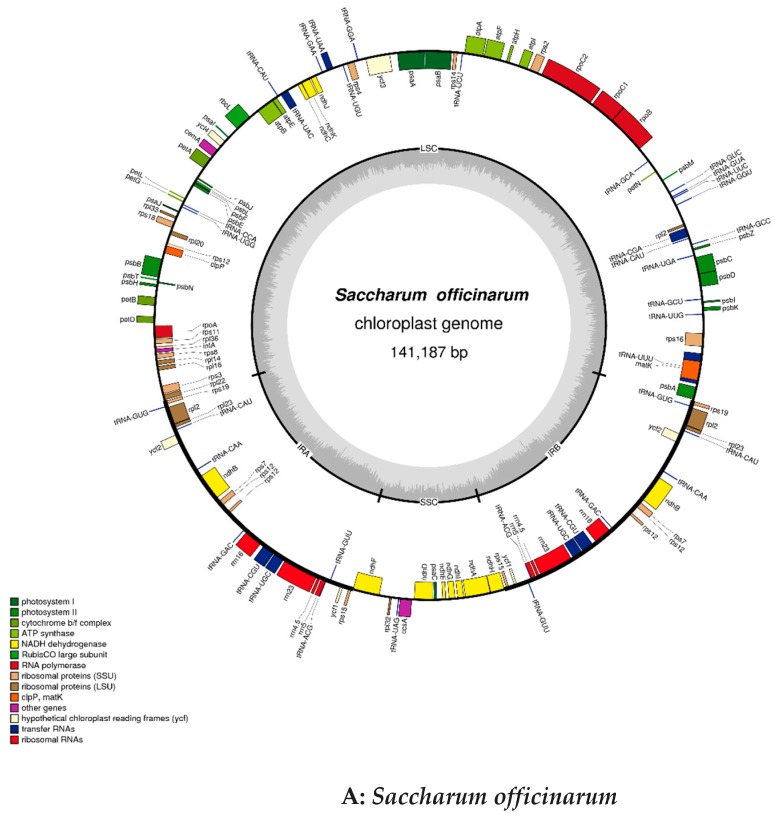

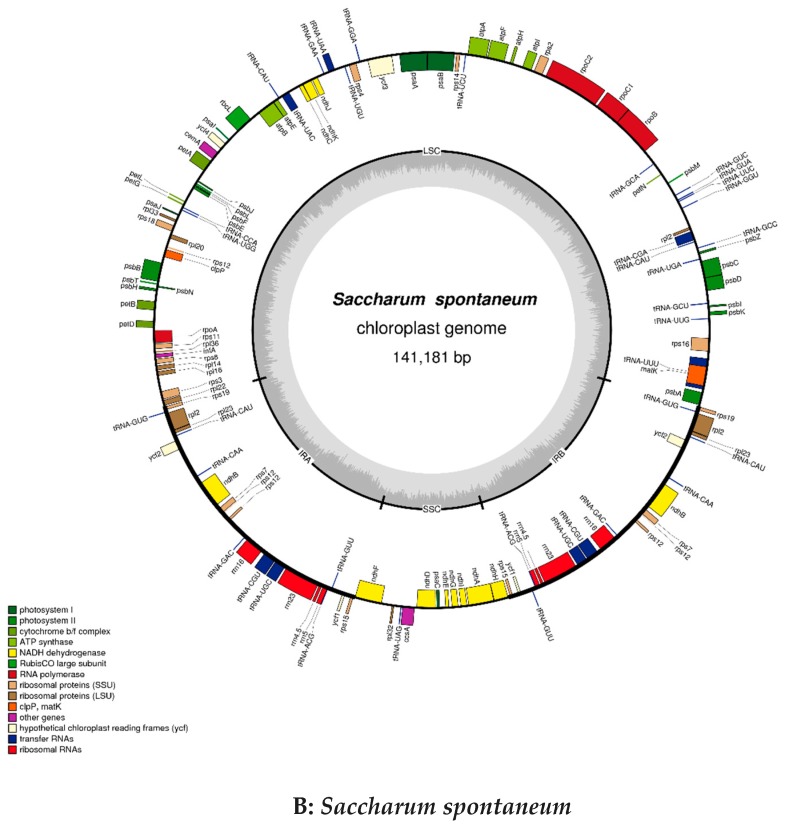
Gene maps of the complete chloroplast genome of *Saccharum officinarum* (**A**) and *S. spontaneum* (**B**) generated by software OGDRAW. As indicated in Figure 2, genes located outside of the outer circle are transcribed in the counterclockwise direction, while those located inside are transcribed in the clockwise direction. Different functional gene groups are represented in color codes. In addition, the variations of GC and AT content are indicated by lighter gray and darker gray plot in the inner circle, respectively.

The size of the cp genome for *S. officinarum* is 141,187 bp, and that is 141,181 bp for *S. spontaneum*, while the GC content (38.44%) is completely the same. The coverage map for assembly chloroplast sequence genomes were showed in Figure 1, which can help to distinguish the coding genes (CDS), tRNA, rRNA, the coverage depth and GC content of the genome. The gene maps of the complete circle chloroplast genomes of *S. officinarum* (A) and *S. spontaneum* (B) are shown in Figure 2. In the cp genome of *S. officinarum*, there is a typical tetrad structure, including the sequences of paired IRA and IRB, which encode in opposite directions, and so is in the species *S. spontaneum*. In addition, there are large and small single copy regions in both cp genomes. The length of the large single copy (LSC) region is 83,065 bp with base sequence position from 1 to 83,065, the small single copy (SSC) region is 12,544 bp (from position 105,855 to 118,398) and both the two inverted repeat (IR) regions are 22,789 bp in *S. officinarum*, of which IRa from position 118,399 to 141,187, and IRb from position 83,066 to 105,854. Among these regions, LSC and IR are 18 bp larger and 6 bp shorter than those in *S. spontaneum* (83,047 bp and 22,795 bp), respectively. Gene annotation showed that there were 134 annotated genes in cp genome of *S. officinarum* and *S. spontaneum*, which is one less than that in cp genome of *Saccharum* hybrid cultivar RB867515 (135) [29]. The gene numbers for all of the mRNA/protein coding genes (CDS) (88), tRNA (38) and rRNA (8) are same in two different species, with 40 duplicated genes in the IR region, while both lacking one tRNA gene compared to the modern cultivar RB867515 (39) [29]. In addition, the unique gene numbers of CDS, tRNA and rRNA are same in both species. Among the CDS genes, there are 12 genes have introns in both species, of which only one gene *ycf3* with two introns, the other 11 genes including *atpF, ndhA, ndhB, rpl2, rps16, trnL-UUU*, *trnS-CGA*, *trnL-UAA*, *trnA-UGC*, *trnV-UAC*, *trnT-CGU* with one intron. Additionally, there are two introns in the gene *rps12* but with a trans-splicing, and thus not counted in our statistic in this study, due to the start sites of both introns are located in the IR region, in spite of sometimes the aforementioned introns indeed having been counted, such as in the study of *Mikania* [7]. Exons existed in all 134 genes, of which most (116) have only one-, 14 genes have two-, and only three genes (*ycf3* and two *rps12*) have three exons. In the IR region, there are 20 genes with two or more copies: *ndhB, rpl2, rpl23, rps12, rps15, rps19, rps7, rrn16, rrn23, rrn4.5, rrn5, tRNA-ACG, tRNA-CAA, tRNA-CAU, tRNA-CAU, tRNA-CGU, tRNA-GAC, tRNA-GUG, tRNA-GUU, tRNA-UGC, ycf1* and *ycf2*, of which the gene *rpl2* with three copies and *tRNA-CAU* with four copies. These characteristics and the number of exons and introns are same in two different species while the positions in the cp genome are divergent for most of exons and introns, in spite of the lengths are same for vast majority, and only two introns with different lengths. Of which, two introns with different length were located in the gene *ycf3*: 780 bp and 736 bp in size for *S. officinarum*, while 754 bp and 737 bp in size for *S. spontaneum*, respectively. Additionally, the intron in *tRNA-UUU* of *S. officinarum* is 2475 bp in size, one base-pairs less compared to that in *S. spontaneum*. Both species have four different types of hypothetical or putative chloroplast genes, including five genes (two *ycf1*, two *ycf2* and one *ycf3* gene) existed in the IR region and one gene *psbJ* existed in the other region. Besides, there is one undefined function gene *rpl2* in two species. Details were presented in supplementary Table S1 (*Saccharum*_*officinarum*.exon.intron.stat) and Table S2 (*Saccharum*_*spontaneum*.exon.intron.stat). The coding protein genes (88) can be divided into four categories: (1) self-replicating genes; (2) photosynthesis genes including light systems I and II, adenosine-triphosphate (ATP) synthase, a cytochrome b6/f protein complex and other biosynthesis genes, such as cytochrome related genes; (3) other genes, including those genes related to biosynthesis; (4) Unknown function protein-coding gene. Details of the gene contents, including gene family, their functions and gene names, in the cp genomes of two sugarcane ancestors were presented in Table 1.

### 2.3. The Collinear Analysis

**Figure 3:**
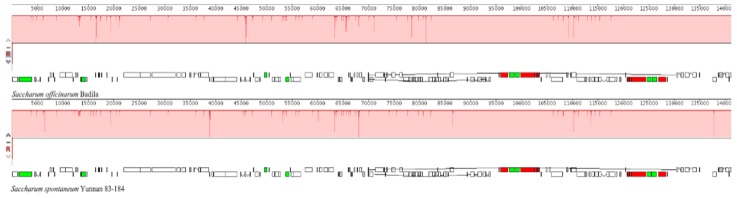
Colinear analysis of chloroplast genomes between two sugarcane ancestors *Saccharum officinarum* and *S. spontaneum*. Color bands represent genes, and different colors represent different blocks. Blocks with the same color between different genes represent homologous regions. Within each block, Mauve software draws similar profiles of genome sequences, and the height of similar profile corresponds to the average conservative level of the sequence region of the genome. Two rows of small blocks below the color band represent genes. The top one is on the positive chain, and the below one is on the complementary chain. Of which, the white block represents the CDS, a thin line inside the white blocks denotes intron. Green and red blocks represent tRNA and rRNA, respectively.

The collinear analysis by Mauve software revealed the highly conserved structures of two sugarcane ancestors, thus the high collinearity was observed between *S. officinarum* var. Badila and *S. spontaneum* var. Yunnan 83-184, no matter which types of genes. However, there still are some sites containing insertions and deletions in their cp genomes (Figure 3). For example, one CDS gene near the site of 20,000, there appeared deletions in size in *S. spontaneum*, while two genes between the sites of 50,000 and 55,000 appeared insertions in *S. spontaneum*. It is interesting to note that two CDS genes between the sites of 65,000 and 70,000 on the positive chain appeared to be contrary in these two species when referring to the size, which is one deleted while the other inserted.

### 2.4. Analysis of Highly Variable Regions and Base Substitutions in CDS Genes

**Figure 4:**
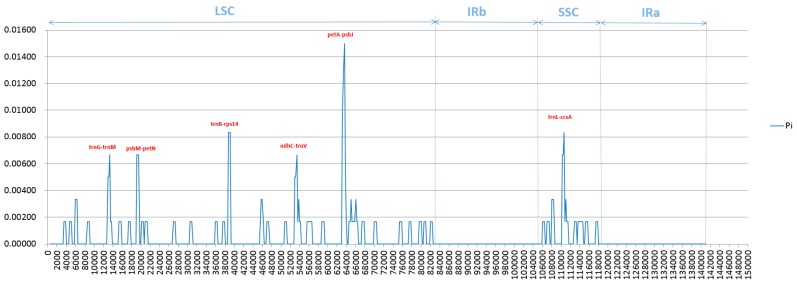
Nucleotide diversity (Pi) based on sliding window analysis in the aligned complete chloroplast genomes of *Saccharum officinarum* and *S. spontaneum*. Performing sliding window analysis with: window length 600 bp, step size 200 bp.

In spite of extreme similarities in the structure and organization in cp genomes of two sugarcane ancestors *S. officinarum* and *S. spontaneum*, the divergences may also exist in the noncoding regions, especially in the regions of intergenic sequence (IGS). Thus, we further investigated the level of divergence by analysis of nucleotide variability (Pi). In the aligned cp genomes of two different species, Pi obtained by sliding window analysis indicated the locations of the variation occurring. The values of Pi range from 0.000 to 0.01500, and those with the percentage of variation higher than 0.00600 (Pi > 0.006) were marked out from a total of 129 divergent genes (Figure 4). According to this Pi value, six of the most variable sites were detected between the two species, of which five in the LSC region, namely *trnG-trnM*, *psbM-petN*, *trnR-rps14*, *ndhC-trnV* and *petA-psbJ*, together with one site named *trnL-ccsA* in the SSC region, while non-site meets the standard in the IR region, indicating the very conservative IRs in both genomes. Additionally, the percentages of variation among the divergent genes are 0.00167, and the gene of *petA-psbJ* in the LSC region was found to have the highest divergent value of 0.01500.

**Figure 5:**
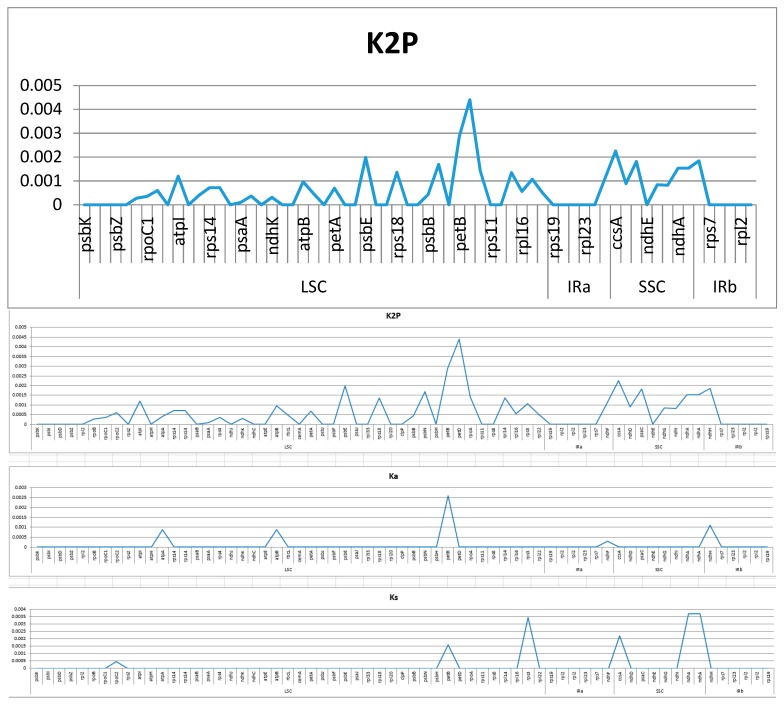
The K2P, Ka and Ks values of the 65 protein-coding genes in high divergent regions from two sugarcane ancestors of *Saccharum officinarum* and *S. spontaneum*.

A total of 65 protein genes shared between two sugarcane ancestors were used to estimate average genetic distances. Their distances vary from 0.00000 to 0.00440, with the average value of 0.00058 (Figure 5). Of these, there are twenty-three genes with greater divergence than average genetic distance (0.00058) in the overall. Of which, *petD* located in the LSC presents the highest level of divergence (0.00440), followed by *petB* (0.00288) which also located in the LSC, and the top third is *ccsA* (0.00225) located in the SSC. Among them, a total of 32 genes showed to be zero referring to genetic distance: 21 in the LSC, each five in the IRa and IRb, and one (*ndhE*) in the SSC. To estimate the evolution pressure on the specific coding protein gene, we carried out an analysis on nonsynonymous (Ka) and synonymous (Ks) substitution rates, of which synonymous mutation is termed as the mutation of nucleotide without resulting in the corresponding changes of the amino acid sequence, while contrary for the nonsynonymous mutations. In this study, among 65 shared protein genes, the Ka values vary from 0 to 0.0026, with a total average value of 0.0001, and the Ks values range from 0 to 0.00345, with a total average value of 0.00023. To investigate the selective pressure indicated by the value of Ka/Ks on these genes in these two species, the values of Ka/Ks were calculated (Figure 5). According to the standards of criteria: neutral (Ka/Ks = l), positively selected (Ka/Ks > 1), and purifying selection (Ka/Ks < 1), the synonymous mutations are believed to be subjected to the natural selection, while contrary for the nonsynonymous mutations. Abide this rule and compared to *S. officinarum*, the genes of *rpoC2* and *rps3* located in the LSC region, together with the genes of *ccsA*, *ndhA* and *ndhA* located in the SSC region, were subject to very strong purifying selection in *S. spontaneum* due to all of their Ka/Ks values being zero, while only *petB* in the LSC region was subject to positive selection. The results indicate that the chloroplast genomes of two sugarcane ancestors have been affected by different environmental pressures during evolution, which may result in the differences in their cp genomes. Genes with Ka/Ks values equal to zero include *psbI*, *atpH*, and *psaC*, indicating that these genes are under very strong purifying selection.

In order to get the detailed information of base substitutions in CDS genes, single nucleotide polymorphism (SNP) locus identified in chloroplast genomes was carried out. An alignment of the cp genomes indicated that their sequences are highly conserved in two sugarcane ancestors, of which only 24 single nucleotide polymorphic sites identified in 16 genes among all 88 CDS genes in *S. spontaneum* when compared to *S. officinarum* (Table 2). These genes are *rpoC1*, *rpoC2*, *atpA*, *ycf3*, *atpB*, *psbE*, *rpoA*, *rpl14*, *rps3*, *ndhB*, *ndhF*, *ccsA*, *ndhD*, *ndhA*, *ndhH* and *ndhB*, of which the gene *ndhB* has two copies. More than one locus presented in four genes (*atpB*, *ndhB*, *ndhF* and *ccsA*), of which *ndhB* have two copies, and five SNP sites exist in *ccsA*. Among the 24 SNP loci, most (16) appeared to be the nonsynonymous substitutions due to the mutation of a single base resulting in the change of amino acids, and only eight SNP loci belong to the synonymous substitutions due to the unchanged of amino acids when base mutation happening. The codons corresponding to the above SNP loci and amino acids in *S. officinarum* and *S. spontaneum* were listed in Table 2. They contain 10 transversions (Tvs) and 10 transitions (Ts) which including six Ts between A and G, four between C and T, and thus giving a ratio of 1:1 for Tv to Ts. Additionally, four codons contain both substitutions, i.e., transversions and transitions.

### 2.5. Coding Capacity of Protein Coding Genes (PCGs) and Relative Synonymous Codon Usage (RSCU)

There is a total of 19,994 codons, which represents the coding capacity of protein coding genes, in cp genome of *S. officinarum*, and 20,436 codons in *S. spontaneum*, indicating the stronger coding capacity in the wild species of *S. spontaneum*, while both coding the same number (21) of different types of amino acids. The amino acids, codons and their corresponding numbers, and relative synonymous codon usage (RSCU) were presented in Table 3. Among them, the most abundant amino acid is leucine, with number of 2175 (10.88% of total) and 2228 (10.90% of total) codons, followed by isoleucine, with number of 1639 and 1671 codons in *S. officinarum* and *S. spontaneum*, respectively. The lowest number is cysteine, with only 221 (1.105%) and 224 (1.096%) codons in *S. officinarum* and *S. spontaneum*, respectively. In general, the codon preference is similar in both species, while within species, there is different preference. The preferred codon is AUG encoding amino acid methionine (Met) with 2.961% RSCU in both species, followed by UUA encoding leucine with 1.992% and 1.966% RSCU in *S. officinarum* and *S. spontaneum*, respectively, and GCU, UAU, ACU, UCU, AGA, UUA, GAU, GGA CCU were listed at the third level. On the contrary, the lowest frequency codon is AUA with 0.013%, followed by GUG with 0.026% in both species. Interestingly, both AUA and GUG encode Met. 

### 2.6. Analysis on IR Junctions Proline, Serine, Glutamine, Glycine, Alanine and/or Asparagine

**Figure 6:**
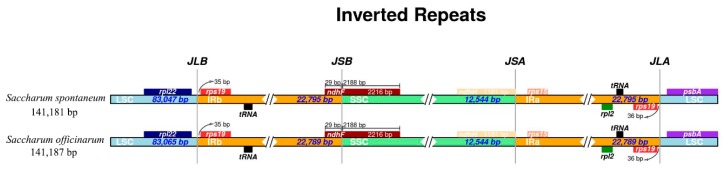
Comparison the junctions of the large single copy (LSC), small single copy (SSC) and inverted repeats (IRs) in chloroplast genomes of *Saccharum officinarum* and *S. spontaneum.* Among them, *JLB* stands for the joint of LSC/ IRb, *JSB* for the joint of SSC/ IRb, *JSA* for the jint of SSC/ IRa, and *JLA* for the joint of LSC/IRa.

The IR region is considered to be relatively conserved and exists four boundaries: IRa/LSC, IRa/SSC, IRb/LSC and IRb/SSC in plant cp genome, while border region contraction and expansion are found to be common and important in the process of evolution, which is the main reason of variation of angiosperm-plant cp genome length [43,44]. In current study, the IR boundaries in two sugarcane ancestor species were compared in detail, and presented in Figure 6. There are several genes showed to be contracted at the boundaries, such as: (1) the genes *rpl22* and *rps19* at the IRb/LSC border contracted 58 bp and 35 bp, but located in the LSC and IRb regions, respectively; (2) another *rps19* with 36 bp contraction at the IRa/LSC border but located in the IRa region, and *psbA* located in LSC region with 90 bp contraction at the IRa/LSC border, together with the gene *rps15* at the IRa/SSC border with 153 bp contraction. On the contrary, there also appeared the gene with the characteristic of expansion, such as the gene *ndhF* at the IRb/SSC border, which located in the SSC region but expanded 29 bp to the IRb region. In addition, the gene *ndhH* at IRa/SSC border without contraction or expansion. In a word, there are several genes with the characteristics of contraction and expansion, while without differences between *S. officinarum* and *S. spontaneum.* Besides, the *ycf1* gene, which is traditionally regarded as the hypothetical gene, has been reported to be necessary for plant viability in the *Arabidopsis* recently [45]. In this study, two species S. *officinarum* and *S. spontaneum* are also observed presenting two *ycf*1 genes in their cp genomes: one contraction at the IRb/SSC boundary and located in the IRb region, another at the IRa/SSC border but located in the IRa region.

### 2.7. Repeat Structure Analysis

When referring to simple sequence repeats (SSRs), different performance parameters can result in different numbers of SSRs detected, strict parameters result in lower amount of SSRs. We performed three different definitions for SSR search, and the results were as follows. When following the stricter performance parameters (unit_size / min_repeats): 1/10 (mononucleotides ≥ 10 nt), 2/6 (dinucleotides ≥ 6 repeats), 3/5, 4/5, 5/5, and 6/5, only 30 SSRs were identified in the cp genome sequences of *S. officinarum*, and all are mononucleotides repeats with the longest repeats 14 except one sequence containing two SSR presenting in compound formation ‘(T)10ctctccta(T)10′ with 28 bp in size (Table 4). In *S. spontaneum*, the results showed to be some differences: one more SSRs (32) identified, one sequence containing two SSR, and two presenting in compound formation. These two compound formations are ‘(A)10ggaactatgattcatactcactatttagacctcgcaaccagactg(A)10′ with 65 bp in size, and ‘(T)10ctctccta(T)10′ with 28 bp in length (Table 4). In addition, the T-repeat unit was the most abundant in both species, with number of 20 for *S. officinarum* and 22 for *S. spontaneum*, respectively. Whilst, the highest frequency of classified repeat types was A/T (considering sequence complementary), with the number of 29 and 31 in *S. officinarum* and *S. spontaneum*, respectively, which is similar to the previous report [46], while both have only one G repeat.

When according to the parameters used in sugarcane by Melotto-Passarin et al. (2011) but with modifications, only 190 SSRs were identified, and the most abundant repeats were mononucleotide repeats (128), followed by tri- (47), tetra- (9), di- (5) and penta- (1) in *S. spontaneum*, with only one sequence containing more than one SSR (2) and three SSRs presenting in compound formations: (TATAA)3ttaat(ATA)3, (A)9t(A)8, and (T)10ctctccta(T)10 (supplementary Tables S3 and S5). In *S. officinarum*, the situations have some differences in spite of being similar in sum: one less SSR (189), the most abundant repeat unit is mononucleotide (126), followed by tri- (47), one sequences containing more than one SSR (2) and three SSRs presenting in compound formation but only one showed to be divergent in size, i.e., (A)9t(A)11 with size 21 bp, though the position for most SSRs were different (supplementary Tables S4 and S5). Detailed information about SSR type, SSR sequence, size, start and end positions of the aforementioned SSRs in cp genome is presented in supplementary excel Table S5. Additionally, if a little less strict parameters were performed, i.e., 3rd set of parameters, there were a total of 477 SSRs from *S. officinarum* were identified, with only one sequences containing more than one SSR and four SSRs presenting in compound formation. While, in *S. spontaneum*, one less SSRs (476) are identified and five SSRs present in compound formation. According to the above definition, the most abundant repeats were penta-nucleotides, followed by hexa-nucleotides in both species. 

### 2.8. Photosynthetic Ability Analysis

The chlorophyll relative content (SPAD) and chlorophyll fluorescence parameter Fv/Fm reflect the photosynthesis ability of species. The average SPAD was 29.25 for *S. officinarum* before cold stress, and 28.02 and 27.73 under cold stress for 3 days and 7 days, and 23.37 after removing from incubator to the field for 10 days recovery, while for *S. spontaneum*, the average values were 45.50, 41.42, 40.42 and 39.48, respectively. The significantly higher of SPAD values were found in *S. spontaneum* than those in *S. officinarum*, no matter the measurement is performed in control conditions or at the early (3 days) or late time (7 days) under cold stress, and so was after cold stress removing. A significant difference was also observed between two species at recovering stage after stress removing. In addition, unobvious differences were observed between the values measured in early (3 days) and late (7 days) time under cold stress and during recovery in *S. spontaneum*, though obvious differences were observed between the control conditions and the aforementioned conditions, which indicating its quick response to the environmental stress. However, the situation was different in *S. officinarum*: unobvious decrease of SPAD value between control conditions and cold stress, while significant decrease during recovery compared to that in the control conditions and in the cold stress, which indicating its slow response to environmental stress and resulting in impaired recovery. These results reflect that the wild species of *S. spontaneum* has stronger photosynthetic capacity than that of *S. officinarum* and is more tolerant to cold stress than that of *S. officinarum*. When referred to chlorophyll fluorescence parameters, the maximal photochemical efficiency in two sugarcane ancestors was estimated (Table 5). The average value of Fv/Fm, i.e., the ratio of the real-time fluorescence vs. maximum fluorescence, was 0.364 ± 0.152 in *S. spontaneum*, which was significantly higher than that (0.194 ± 0.096) in *S. officinarum* after exposure to cold environments for 7 days. In addition, after relieving cold stress and cultured in the control conditions for 10 days, chlorophyll fluorescence could almost not be detected on the treated plants due to the most investigated leaves (5/6) appeared to be zero referring to the Fv/Fm value, which may be caused by excessive low temperature stress and resulted in impaired recovery even if the cold stress having been removed. The situation for this photosynthetic parameter value was similar in both investigated species. However, surprisingly, we found several new tillers appeared in *S. spontaneum* after for two additional weeks cultured in the control conditions, while no tillers appeared in *S. officinarum* (Figure S2), suggesting again the stronger tolerance to cold stress and stronger growth compensation ability of *S. spontaneum* than that of *S. officinarum*.

## 3. Discussion

This study provides the new data obtained from two modern sugarcane ancestors *S. officinarum* (2n = 80, 8×) and *S. spontaneum* (2n = 80, 10×), and firstly presents the detailed comparison of the complete cp genomes between these two crucial species with the same chromosome number but a little different in ploidy, which may aid to reduce the divergences caused by the number of their chromosomes when referring to the investigation on the expanding function of cold tolerance, as we know that the number of chromosomes varies greatly in *S. officinarum* and *S. spontaneum* [24], in spite of the non-existence of chromosomes in chloroplasts. Additionally, *S. spontaneum* accession Yunnan 83-184 has been widely used in the program of basic hybridization to obtain innovative breeding materials and cross parents due to its strong cold tolerance, vigor and excellent resistance to sugarcane diseases, and Badila, in spite of widely cultivated in China as chewing cane, is one of the several limited original clones of *S. officinarum*. It has been successfully induced to flowering in recent ten years, and used widely in basic hybridization. In addition, this study firstly presents the data associated to the photosynthetic capacity, offers an opportunity to further understand the relations of genes and physiological characters. Besides, the detailed information of sequences of CDS genes can provide the basis for investigation of gene expression in chloroplast under environmental stresses, and SSRs and SNP loci can be used to develop the chloroplast markers to track the genetic background during their utilization in sugarcane breeding. Besides, the polymorphic sites may also be used as the basis for development of the molecular markers associated to interesting phenotypic traits, such as resistance, by detection of the SNP loci among the population derived from the hybridization of *S. officinarum* and *S. spontaneum* due to the limited utilization of accessions in two sugarcane ancestor species in modern sugarcane improvement.

Based on the comparison of the cp genomes among six important sugarcane hybrids, including NCo310, SP80-3280, Q155, RB867515, Q165 and RB72454, and two ancestor species *S. officinarum* (Badila) and *S. spontaneum* (Yunnan 83-184), highly conserved genome structures were observed, which are similar to other plant species in different genera [2,3,29,47] or in different families [7,11,27,48,49], while the length is divergent among different accessions. Based on the previous reports [6,27,28,29], GenBank information (Accessions:NC_035224.1; LS975131.1; LN849912.1; LN849914.1; LN896359.1) and two species sequenced in this study, the complete cp genome sequences from 141,151 bp to 141,348 bp among these 11 accessions including the above six sugarcane hybrids and five sugarcane ancestor accessions (Table 6). The length for SSC region is exactly the same except SP80-3280 with additional two base-pairs, which was sequenced in early time [28]. However, diversification mainly appears in the length of LSC, from 83,017 bp to 83,226 bp. The maximum difference in length existed in the species *S. spontaneum* (from 83,047 to 83,226 bp), which may come from much diversification in ploidy (2n = 4× to 12×) and in the number of chromosomes (2n = 40–128), in spite of chloroplast without containing the chromosome, followed by another ancestor *S. officinarum*, with 83,065 bp for Badila and 83,042 bp for IJ76-514. Merely, most modern sugarcane varieties share the same length in the LSC region except for RB72454 with 30 reduced base pairs.

In addition to the divergences in length, differences are also present in gene content of cp genome. There are 38 tRNA genes presented in cp genomes of Badila and Yunnan 83-184, while there is 39 in RB867515 [29], and certainly differing more from the early report of sugarcane cp genome, such as 116 identified genes including 82 CDS, four rRNA and 30 tRNA genes in sugarcane hybrid SP80-3280 [28], which may be due to the difference in sequencing technique. Additionally, the number of the identified genes and duplicated genes in the cp genomes were divergent more in different genera and families. Additionally, as if it is not always appeared to have a positive relation between the size of cp genome and the gene number, in spite of most of them do. In this study, two sugarcane ancestors in genus *Saccharum* have 134 genes identified with 141,187 bp or 141,181 bp in size, and 20 duplicated genes, while *Triticum turgidum subsp. Durum* belongs to the same family (Gramineae), which has 135 genes with a shorter length (135,948 bp) [15] than those in sugarcane clones. This phenomenon is also observed between *T. timopheevii* cultivar TA944 (KJ614409) with 136,124 bp in size while only 85 annotated genes, and *T. timopheevii* cultivar Tim01 (NC_024764) with 136,157 bp in size with 125 annotated genes [15]. Different situation was found between *Hordeum vulgare* and sugarcane, and *Sorghum bicolor* and sugarcane, both groups have the characteristics of less annotated genes (131) with the smaller length (136,462 bp and 140,754 bp) [50], and thus suggesting a positive relation between the length of cp genome and the gene number. A similar situation was also found in *Ipomoea* L. from Convolvulaceae [51] and between *Triticum turgidum subsp. Durum* (KM352501) and *T. urartu* (NC_021762) [15].

More divergences were observed when referred to the base substitution events. In this study, we find 24 SNP loci in *S. spontaneum* when reference to *S. officinarum*, which was different from the findings of Vidigal et al. [29], in which sugarcane hybrid RB867515 was identical to Q155, and only four SNPs and one Indel differing from NCo310, six SNPs and two Indels differing from SP80-3280. Even though this rate of base substitutions (0.017%) between *S. spontaneum* and *S. officinarum* is low at the intrageneric level, but higher than that observed by Vidigal et al. [29]. This rate was much lower than the 5940, 6260 and 5992 between *Fagopyrum luojishanense* and each of *F. dibotrys*, *F. esculentum* and *F. tataricum* [46], and the 591 (0.38%) between *Solanum bulbocastanum* and *S. tuberosum* [48], and the 235 (0.15%) between *Mikania micrantha* and *M. cordata* [7] and the 231 (0.15%) between *Machilus balansae* and *M. yunnanensis* [47]. Specifically, the SNPs existed in 72 out of 79 CDS genes among seven *Panax* species [52], with the exception of seven genes: *psaJ*, *psbN*, *rpl23*, *psbF*, *psbL*, *rps18*, and *rps7*. In addition, when referring to SNP density, lower density in IR regions was observed than those in LSC and SSC regions, similar phenomena were observed in the other species, such as in *Panax* [52], *Solanum* [48], and *Machilus* [47], implying that it is a common phenomenon. Among the 24 SNP loci, most (16) appeared to be the nonsynonymous substitutions, and this rate (66.67%) of the nonsynonymous and synonymous substitutions is high. Whilst, the ratio of 1:1 for Tv to Ts between *S. spontaneum* and *S. officinarum* suggests that substitutions occur with unbias, while substitutions occurring with a bias and in favor of transversions was observed between *M. micrantha* and *M. cordata* with the rate of 1:0.74 [7]. The base substitutions identified in the current study may help to understand the phylogeny and population genetics of *Saccharum*, and recognize the real hybrids in sugarcane genetic improvement.

Codons play an important role in the process of transmission of genetic information. The number of total codons for protein coding genes are divergent in different species. There are only 19,994 codons in *S. officinarum* var. Badila and 20,436 in *S. spontaneum* Yunnan 83-184, less than those in *M. micrantha* (26,417) and *M. cordata* (26,414) [7], while coding more CDS genes (88) than that (80) in *M. micrantha* and *M. cordata*, implying the stronger coding capacity. However, the most and the least abundant amino acids are the same in two sugarcane ancestors in Gramineae. They are leucine and cysteine, respectively, and the usage frequency is also high similarity. This high similarity is also observed between different families, such as in *Mikania* (Asteraceae), the most abundant is leucine (10.7%) and the least abundant is cysteine (1.12% and 1.13%) [7] compared to 10.88% and 10.90% for leucine, and 1.105% and 1.096% for cysteine in *S. officinarum* and *S. spontaneum*, respectively, in the current study. This situation is similar in the same family but different genus, such as *M. floridulus* (accession: LN869215.1) in Gramineae [30]. In addition, a codon use preference is common in plant species. In this study, obvious codon use preferences were observed in two sugarcane ancestors *S. officinarum* and *S. spontaneum*, and the most preferred codon is AUG, followed by UUA, and then GCU, UAU, ACU, UCU, AGA, UUA, GAU, GGA and CCU with the similar preferences in both species. Similar opinion of obvious codon use preferences was also found in the previous studies of *Pyrus* [53] and *F. dibotrys* [44]. However, the preferred codons are divergent in different species. For example, the most preferred codon is AUG in our study, while the frequently used codons are ATT, AAA, GAA, AAT and TTT in *Pyrus*, and the most preferred one is ATT [53], indicating that different species have divergent codon preferences, which is formed during the long-term of evolutionary process.

Repeat structures are correlated with rearrangement and recombination of plastome including chloroplast genome. SSRs in cp genome are potentially useful markers for population genetics due to a high variation commonly appeared within the same species [54]. In our study, a large number of repeats were detected in the cp genomes of sugarcane ancestors, and most repeats were located in intergenic regions, which is similar to the species in *Fagopyrum* [44], and most are perfect types with only one to two compound SSRs in either of the three sets of parameters performed in this study, which is similar to the species *M. micrantha* and *M. cordata* [7]. However, performance parameters for searching SSRs determine the number of SSRs detected, and significant difference will be observed after carrying out three sets of parameters. There are 30 limited SSRs with mononucleotide repeats when parameters of 1/10 (mononucleotides ≥ 10 nt), 2/6 (dinucleotides ≥ 6 repeats), 3/5, 4/5, 5/5, and (6/5) were performed, compared to a large number of more than 470 SSRs when less strict parameters were performed. Thus, performance parameter is the most important factor in investigation of SSR, which can be adjusted according to the desired and objective. 

In addition to the crucial function of performing photosynthesis in the chloroplast, it also evolves to extend functions, such as tolerance to environmental stresses of low temperature. In chloroplast, impaired chlorophyll biosynthesis was observed when plants exposed to cold stress, which is attributed to down-regulation of gene expression, protein abundance and enzyme activities [32,55,56,57]. The reduced photosynthesis, caused by low temperature stress, is due to decline in Fv/Fm, PSII, inhibition of electron transport, and consequent declined photophosphorylation and CO_2_ assimilation [58,59,60,61,62,63]. In the current study, compared to *S. officinarum*, the values of SPAD in *S. spontaneum* were always significantly higher no matter they are cultivated in control temperature, exposed to low temperature or during recovery, and so is for Fv/Fm when exposure to low temperature. In addition, during recovery, there are several new strong tillers observed in *S. spontaneum* but no tillers appeared in *S. officinarum*, indicating a strong growth compensation ability in *S. spontaneum,* in spite of the value of Fv/Fm can not be detected in both species, which may result from excessive low temperature stress. However, when compared with the Fv/Fm values obtained in sugarcane under control field conditions, these values observed in both accessions of Badila and Yunnan 83-184 are quite low [64]. For Badila, the Fv/Fm value observed in cold stress in this study is only 27% of its value in control field conditions [64]. It suggests that *S. spontaneum* Yunnan 83-184 exhibits the stronger tolerance to cold stress than *S. officinarum* Badila, and the expanding function to low temperature stress in chloroplast also observed in sugarcane. In addition, the stronger tolerance to low temperature environmental stress of *S. spontaneum* can also reflect in quick response to cold stress than that in *S. officinarum*, and thus results in less damage. Merely, low temperature still has a serious impact on chloroplast of *S. spontaneum* and *S. officinarum*, which results in obvious decrease of the Fv/Fm value but unobvious for SPAD during cold stress. 

## 4. Materials and Methods

### 4.1. Plant Material, Sample Collection and DNA Preparation

Considering the complexity and diversity of ploidy and chromosome number of *Saccharum officinarum* and *S. spontaneum*, accessions from these two sugarcane ancestor species, used for analysis of chloroplast genome sequence, especially cold tolerance, should have the same or at least not much difference in ploidy and chromosome number, because both factors have great influence on phenotypes, including biomass. Thus, two accessions Badila and Yunnan 83-184 from the aforementioned ancestor species were selected for investigation. Young fresh leaves of *Saccharum officinarum* var. Badila (chromosome number 2n = 80, ploidy 8×) and *S. spontaneum* Yunnan 83-184 (2n = 80, 10×) were collected from the National Sugarcane Germplasm Resource Garden in Kaiyuan, Yunnan, China. Yunan 83-184 is an important wild type of *S. spontaneum*, which has good performance in drought, cold and salty stress resistance, and growth vigor, and has been widely used in germplasm innovation. Badila is one of the several limited original accessions of *S. officinarum*, which is also popularly used as the cultivar for chewing cane in China for a long time. A modified cetyl-trimethylammonium bromide (CTAB)-based method [65] was used for extracting leaf total DNA. Then, the purified DNA with suitable quality was stored at −80 °C for further use.

### 4.2. DNA Sequencing and Genome Assembly

The purified DNA was used for the construction of chloroplast DNA libraries. Illumina HiSeq 2500 was used to generate raw sequence reads for this project. Since the DNA sample for sequencing are a mixture from nucleus and organelles, the cpDNA sequences needed to be separated from the original raw reads. After removing adaptors and low-quality reads (Q ≤ 30), clean reads were assembled by Genome Assembler SPAdes (version 3.13.1) (cab.spbu.ru/software/spades/v) to get the seed sequences, then kmer (kmer = “55, 87, 121”) iterative extend seed, and then using SSPACE to scaffold contigs. The software GapFiller v2.1.1 (https://jaist.dl.sourceforge.net/project/gapfiller/v2.1.1/gapfiller-2.1.1.tar.gz) was used for filling up the gaps. Before assembling, chloroplast DNA (cpDNA) sequences were drawn by the method of Bowtie2 version 2.2.4 by Ben Langmead (langmea@cs.jhu.edu, www.cs.jhu.edu/~langmea) from the paired-end reads by comparing to the known database of plant chloroplast genomes constructed by the Genepeer Biotechnology Company (Nanjing, China), in which the cp genome sequences were downloaded from NCBI, and then these reads were used to assemble the cp genomes. Assembled contigs were subjected to BLAST against the existing complete cp sequence of *S. spontaneum* IJ76-287 (accession number: LS975131.1), which was also used as the reference cp genome to assemble into the complete circular cp genome sequence. The following method was occupied for filling up the gaps between contigs, and described as: using BLAST to map the raw reads onto both ends of the assembled contigs (step one), and then scaffolds and joining overlapping reads to elongate the contig (step two). The above two steps were repeatedly carried out till all the gaps between contigs were filled up. Sequence assembly of clean reads was carried out according to the chloroplast genome sequences of reference species. Gene annotation, RSCU analysis, cpSSR analysis, statistical analysis of K2P, Ka and Ks, and co-linear analysis of two sequenced species were conducted based on the assembly results of cp sequences.

### 4.3. Comparison of Complete cp Genomes, Genomic Annotation and Analysis

BioEdit v7.2.5 (https://download.informer.com/win-1191694432-75407f8f-6a559e4d/bioedit.zip) was occupied to investigate variations in the cp genome of *S. officinarum* and *S. spontaneum*. The LSC/IRB/SSC/IRA boundary regions were compared by BLASTN of homologous sequences. The software of BioEdit v7.2.5 was used to extract the sequences of coding, rRNA, tRNA, intronic and intergenic sequences, and the same for analysis of GC content. Prodigal Software v2.6.3 (https://github.com/hyattpd/Prodigal) and Software Maker v2.31.10 (http://www.yandell-lab.org/software/maker.html) were used for gene prediction, and the online tool DOGMA was used to assist verification. Additionally, an investigation of synonymous codon usage was accomplished by CodonW v1.4.2. DOGMA (http://dogma.ccbb.utexas.edu/ http://dogma.ccbb.utexas.edu, the Dual Organellar GenoMe Annotator), while start codons, stop codons, intron and exon positions were manually adjusted by means of comparison with homologous genes from *S. spontaneum* cp genomes (LS975131.1). ORF Finder (http://www.ncbi.nlm.nih.gov/gorf/orfig.cgi) was used to determine the open reading frames (ORF). The software Aragorn was used to predict tRNA, and Hmmer for rRNA genes [66]. OGDraw (http://ogdraw.mpimp-golm. mpg.de/) was used to generate the circular genome map, and the sequences of complete cp genome were deposited in GenBank. In addition, the alignment between the chloroplast genomes of *S. officinarum* and *S. spontaneum* was obtained by Clustalw v2.1 (http://www.clustal.org/download/current/clustalw-2.1.tar.gz). Using software DnaSP v5.0 (http://www.ub.edu/dnasp/). to perform an analysis of sliding window by searching of nucleotide diversity (Pi) with the standards: 600-bp window length and 200-bp step size. Clustalw v2.1 was used for multiple sequence alignment, and sequence divergences of pair-wise were estimated by Kimura’s two-parameter model [67]. KaKs_Calculator2.0 (https://sourceforge.net/projects/kakscalculator2/) [68] was used for estimating Ka and Ks. Besides, BLASTN was used to investigate the boundary regions.

### 4.4. Repeat Structure Analysis

In the cp genomes of *S. officinarum* and *S. spontaneum*, microsatellites or simple sequence repeats (SSRs) among tandem repeat structures were identified by MISA (MIcroSAtellite identification tool) (http://pgrc.ipk-gatersleben.de/misa/). In order to compare the differences, we performed SSR identification following three different definitions (unit_size/ minimum number of repeats), which as follows: (1) 1st set of parameters: including at least ten for mononucleotides (1/10), six repeat units for dinucleotides (2/6), five for tri-(3/5), tetra-(4/5), penta-(5/5), and hexa-nucleotides (6/5); (2) 2nd set of parameters based on the report of Melotto-Passarin et al. [69] but with modification, and the definition of microsatellites (unit size/minimum number of repeats): (1/8) (2/5) (3/3) (4/3) (5/3) (6/3); 3rd set of performance parameters, similar to Raubeson et al. [43], with 10, or more than 10, nucleotides belonging to a less strict parameters for tetra-, penta-, and hexa- nucleotide repeat units: (1/10), (2/5), (3/4), (4/3), (5/2), and (6/2).

### 4.5. Photosynthetic Parameter Measurement and Statistical Analysis

Stalks of *S. officinarum* and *S. spontaneum* accessions with single bud were cultivated in pots with 50 cm (diameter) × 50 cm (high). Six-month-old plants with seven full-expanded leaves were used to investigate the chlorophyll content, chlorophyll fluorescence parameters and the effects of cold stress on these two parameters. Six biological replicates were set, and one pot for one replicate containing two plants. The middle part of the +1 leaf on one plantlet from each pot was used for measurement. Cold stress was carried out in the low temperature light incubator (LGX-1200D-LED, PRANDT Instrument Co., Ltd., Hangzhou, China) under 4 °C with 12 h light/12 h darkness, and light intensity is same (25,000 Lux), and stopped after 7 days, followed by removing pots to field. A SPAD-502 Plus (Konica Minolta Sensing, Inc., Osaka, Japan) was used to measure the chlorophyll relative content on six plants reference to the previous report [70]. A system of IMAGING-PAM fluorometer (Walz, Effeltrich, Germany) was used to measure the chlorophyll fluorescence parameters on the same six plants and the same leaves, which previously used to investigate the SPAD values according to the method described by Su et al. [71], following a dark adaptation for 2 h. The measurement of chlorophyll relative content was performed before cold stress, culturing for 3 days and 7 days under low temperature, and at 10 days after removing to field for recovering growth. Additionally, the software SPSS 19.0 system (SPSS IBM, Somers, NY, USA) was used to perform the test of significance of difference of physiological data SPAD and Fv/Fm under Duncan’s significant difference test at the *p* < 0.05 level.5. Conclusion

The length of complete cp genome containing a quadripartite structure is 141,187 bp for *S. officinarum*, which is only six bp larger than that of *S. spontaneum*. Its LSC and IR are, respectively, 18 bp larger and 6 bp shorter than those of *S. spontaneum*, while GC contents are same (38.44%). They have the same number of the annotated genes, duplicated genes, and introns (without counting a trans-splicing gene *rps12*)*,* which indicated the high conservativeness in their cp genome sequences. However, each species has its unique genes, and most (16/24) of the detected SNPs are nonsynonymous substitutions in *S. spontaneum* referenced to *S. officinarum*, of which *ccsA* has four SNP loci. In addition, a ratio of 1:1 for Tv (10) to Ts (10) was observed, and four codons contain both substitutions. IR junction analysis indicates that both two ancestors have the same number (5) of contraction genes, and only *ndhF* at the IRb/SSC border with characteristic of expansion. There are more codons for coding amino acids in *S. spontaneum* than that in *S. officinarum,* and there is different preference within species, while both have obvious codon preference and the codons with highest- and lowest-frequency are the same, and so are the most abundant and least amino acids. A batch of SSRs with different repeat types were identified, which could be used to infer the population genetic structure. The wild species *S. spontaneum* exhibits much stronger photosynthetic ability and cold tolerance, which are reflected by the significant higher SPAD in control conditions, under cold stress and during recovery than those in *S. officinarum*, together with the obvious higher chlorophyll fluorescence parameters Fv/Fm when exposed to low temperature, which can also be indicated by new tillers and implied by quick response to low temperature environments. This study adds our new knowledge of the two sugarcane ancestors and highlights the differences in the cp genomes, photosynthetic ability and cold tolerance between two important species, and provides the clues to promote genetic improvement of photosynthetic ability and cold resistance in sugarcane.

## Figures and Tables

**Table 1:** Gene contents in the chloroplast genomes of *Saccharum officinarum* and *S. spontaneum*.

Functions	Family Genes	List of Genes
Photosynthesis genes	Subunits of photosystem I	*psaA, psaB, psaC, psaI, psaJ, ycf4*
	Subunits of photosystem II	*psbA*, *psbB, psbC, psbD, psbE, psbF, psbH, psbI, psbJ, psbK, psbL, psbM, psbN, psbT, psbZ*
	Subunits of Cytochrome b6/f/complex	*petA, petB, petD, petG, petL, petN*
	Subunits of ATP synthase	*atpA, atpB, atpE, atpF ^1^, atpH, atpI*
	Subunits of Rubisco	*rbcL*
	Subunits of NADH-dehydrogenase	*ndhA*^1^, *ndhB*^1, *^, *ndhC*, *ndhD*, *ndhE*, *ndhF*, *ndhG*, *ndhH*, *ndhI*, *ndhJ*, *ndhK*
Self-replicating genes	Large subunit ribosomal proteins	*rpl2*^1, *^, rpl14, rpl16, rpl20, rpl22, rpl23 ^*^, rpl32, rpl33, rpl36
	Small subunit ribosomal proteins	*rps2, rps3, rps4, rps7, rps8, rps11, rps12^#^, rps14, rps15, rps16 ^1^, rps18, rps19*
	DNA dependent RNA polymerase	*rpoA, B, C1, C2*
	Ribosomal RNA genes	rrn23^*^, rrn4.5^*^, rrn 5^*^, rrn16^*^
	Transfer RNA genes	*trnL-UUU*^1^, *trnG-UUG, trnS-GCU, trnS-UGA, trnG-GCC, trnM-CAU, trnS-CGA*^1^, *trnT-GGU, trnG-UUC, trnT-GUA, trnA-GUC, trnC-GCA, trnA-UCU, trnS-GGA, trnT-UGU, trnL-UAA*^1^, *trnP-GAA, trnV-UAC*^1^, trnM-CAU, trnT-CCA, trnP-UGG, trnH-GUG, trnM-CAU ^*^, trnL-CAA ^*^, trnV-GAC, trnT-CGU^1,*^, *trnA-UGC*^1,*^, trnA-ACG, trnA-GUU ^*^, trnL-UAG, trnA-ACG, trnV-GAC, trnH-GUG
Other genes	Envelop membrane protein	*cemA*
	Translational initiation factor	*infA*
	c-type cytochrome synthesis gene	*ccsA*
	ATP-dependent/Protease	*clpP*
	Maturase	*matK*
Unknown function protein-coding gene		ycf1^*^, *ycf*2 ^*^, *ycf 3*^2^

Notes: * Two gene copies in IRs; # Trans-splicing gene; ^1^ Gene containing a single intron; ^2^ Gene containing two introns.

**Table 2:** SNP loci identified in *Saccharum spontaneum* reference to *S. officinarum*.

Gene Order in cp Genome	Gene Name	Position	Code of Base in Reference	Code in *S. spontaneum*	Code of Amino Acid in Reference	Amino Acids in *S. spontaneum*
13	*rpoC1*	2059	ATA	GTA	I	V
14	*rpoC2*	3228	GGC	GGT	G	G
19	*atpA*	572	GGT	GAT	G	D
23	*ycf3*	300	GCA	GCG	A	A
29	*atpB*	313	ACT	GCT	T	A
29	*atpB*	940	GCC	ACC	A	T
38	*psbE*	57	TAC	TAT	Y	Y
52	*rpoA*	202	GGG	TGG	G	W
57	*rpl14*	335	TTT	TAT	F	Y
59	*rps3*	267	CCC	CCA	P	P
65	*ndhB*	776	GAC	GGA	D	G
65	*ndhB*	777	GAC	GGA	D	G
70	*ndhF*	868	ATA	CTA	I	L
70	*ndhF*	1804	ATC	CTC	I	L
72	*ccsA*	537	TTT	TTA	F	L
72	*ccsA*	538	CTT	AGA	L	R
72	*ccsA*	539	CTT	AGA	L	R
72	*ccsA*	540	CTT	AGA	L	R
72	*ccsA*	939	TAT	TAC	Y	Y
73	*ndhD*	1240	TTA	GTA	L	V
78	*ndhA*	858	TCT	TCC	S	S
79	*ndhH*	793	ATC	GTC	I	V
84	*ndhB*	776	GAC	GGA	D	G
84	*ndhB*	777	GAC	GGA	D	G

**Table 3:** Coding capacity of protein coding genes (PCGs) and relative synonymous codon usage (RSCU).

Amino Acid	Symbol	Codon	*Saccharum officinarum*		*S. spontaneum*	
			Number	RSCU	Number	RSCU
*	Ter	UAA	40	1.579	39	1.520
*	Ter	UAG	19	0.750	19	0.740
*	Ter	UGA	17	0.671	19	0.740
A	Ala	GCA	368	1.198	373	1.192
A	Ala	GCC	189	0.615	194	0.62
A	Ala	GCG	134	0.436	137	0.438
A	Ala	GCU	538	1.751	548	1.751
C	Cys	UGC	55	0.498	55	0.491
C	Cys	UGU	166	1.502	169	1.509
D	Asp	GAC	155	0.437	155	0.433
D	Asp	GAU	555	1.563	561	1.567
E	Glu	GAA	794	1.497	803	1.484
E	Glu	GAG	267	0.503	279	0.516
F	Phe	UUC	403	0.718	422	0.732
F	Phe	UUU	720	1.282	731	1.268
G	Gly	GGA	586	1.570	596	1.567
G	Gly	GGC	151	0.404	150	0.394
G	Gly	GGG	275	0.737	284	0.747
G	Gly	GGU	481	1.289	491	1.291
H	His	CAC	120	0.512	122	0.501
H	His	CAU	349	1.488	365	1.499
I	Ile	AUA	522	0.956	540	0.970
I	Ile	AUC	307	0.562	308	0.553
I	Ile	AUU	810	1.483	823	1.478
K	Lys	AAA	743	1.450	751	1.437
K	Lys	AAG	282	0.550	294	0.563
L	Leu	CUA	324	0.894	338	0.910
L	Leu	CUC	138	0.380	137	0.369
L	Leu	CUG	116	0.320	116	0.313
L	Leu	CUU	476	1.313	500	1.346
L	Leu	UUA	722	1.992	730	1.966
L	Leu	UUG	399	1.100	407	1.096
M	Met	AUA	2	0.013	2	0.013
M	Met	AUG	456	2.961	458	2.961
M	Met	GUG	4	0.026	4	0.026
N	Asn	AAC	208	0.515	218	0.527
N	Asn	AAU	599	1.485	609	1.473
P	Pro	CCA	216	1.013	222	1.026
P	Pro	CCC	208	0.975	212	0.979
P	Pro	CCG	93	0.436	94	0.434
P	Pro	CCU	336	1.576	338	1.561
Q	Gln	CAA	524	1.555	532	1.549
Q	Gln	CAG	150	0.445	155	0.451
R	Arg	AGA	361	1.758	376	1.764
R	Arg	AGG	110	0.536	114	0.535
R	Arg	CGA	273	1.330	285	1.337
R	Arg	CGC	106	0.516	110	0.516
R	Arg	CGG	94	0.458	100	0.469
R	Arg	CGU	288	1.403	294	1.379
S	Ser	AGC	104	0.434	106	0.428
S	Ser	AGU	297	1.241	303	1.225
S	Ser	UCA	235	0.982	237	0.958
S	Ser	UCC	286	1.195	302	1.221
S	Ser	UCG	122	0.510	130	0.526
S	Ser	UCU	392	1.638	406	1.642
T	Thr	ACA	300	1.109	304	1.094
T	Thr	ACC	203	0.750	211	0.759
T	Thr	ACG	133	0.492	137	0.493
T	Thr	ACU	446	1.649	460	1.655
V	Val	GUA	431	1.504	438	1.516
V	Val	GUC	134	0.468	137	0.474
V	Val	GUG	150	0.524	152	0.526
V	Val	GUU	431	1.504	429	1.484
W	Trp	UGG	347	1.000	353	1.000
Y	Tyr	UAC	151	0.411	154	0.410
Y	Tyr	UAU	583	1.589	598	1.590

Notes: * stands for the terminator.

**Table 4:** SSRs identified in chloroplast genome sequences of *Saccharum officinarum* and *S. spontaneum*.

*S. officinarum*				*S. spontaneum*			
SSR	Size/bp	Start Position	End Position	SSR	Size/bp	Start Position	End Position
(A)13	13	3754	3766	(A)15	15	3753	3767
(A)11	11	4114	4124	(A)10	10	4115	4124
(T)10	10	6446	6455	(T)11	11	6112	6122
(A)11	11	7777	7787	(T)14	14	6446	6459
(T)10	10	9056	9065	(A)11	11	7781	7791
(G)10	10	11,003	11,012	(T)10	10	9060	9069
(T)11	11	13,372	13,382	(G)10	10	11,007	11,016
(T)13	13	16,556	16,568	(T)11	11	13,375	13,385
(A)10	10	18,659	18,668	(A)10	10	15,948	15,957
(A)12	12	19,205	19,216	(T)14	14	16,554	16,567
(T)12	12	21,069	21,080	(A)12	12	19,197	19,208
(A)10	10	31,914	31,923	(T)10	10	21,061	21,070
(T)11	11	34,093	34,103	(A)10	10	31,904	31,913
(T)10	10	34,862	34,871	(T)11	11	34,083	34,093
(T)10	10	35,824	35,833	(T)10	10	34,852	34,861
(T)10	10	38,697	38,706	(T)10	10	35,814	35,823
(T)10	10	44,257	44,266	(T)11	11	38,687	38,697
(T)14	14	52,367	52,380	(T)11	11	38,836	38,846
(T)10	10	56,710	56,719	(T)14	14	52,356	52,369
(T)10	10	56,892	56,901	(T)10	10	56,700	56,709
(T)10	10	57,363	57,372	(T)10	10	57,351	57,360
(A)11	11	63,551	63,561	(T)11	11	60,122	60,132
(A)11	11	65,513	65,523	(T)10	10	66,448	66,457
(T)10	10	67,486	67,495	(T)10	10	67,465	67,474
(T)11	11	68,139	68,149	(T)12	12	68,133	68,144
(A)10	10	73,938	73,947	C2	65	73,929	73,993
(T)12	12	78,482	78,493	(T)12	12	78,474	78,485
C1	28	79,044	79,071	C3	28	79,031	79,058
(T)14	14	81,260	81,273	(T)13	13	81,243	81,255
?	?	?	?	(A)10	10	108,278	108,287

Notes: C1, C2, C3 represent the compound formation. Among them, C1: (T)10ctctccta(T)10; C2:(A)10ggaactatgattcatactcactatttagacctcgcaaccagactg(A)10; C3:(T)10ctctccta(T)10.

**Table 5:** The values of SPAD and Fv/Fm measured in two sugarcane ancestors of *Saccharum*.

Investigate Time	SPAD		*p* Value	Fv/Fm	
	*S. officinarum*	*S. spontaneum*		*S. officinarum*	*S. spontaneum*
Before cold stress	29.25 ± 1.50A	45.50 ± 1.40C	0.000008	-	-
Culture at 4 °C for 3 days	28.02 ± 1.10A	41.42 ± 1.88D	0.000013	-	-
Culture at 4 °C for 7 days	27.73 ± 1.16A	40.42 ± 1.25D	0.001401	0.194 ± 0.096A	0.364 ± 0.152B
Removing to field for recovery 10 days	23.37 ± 2.64B	39.48 ± 3.34D	0.000100	0 (5/6 plants)	0 (5/6 plants)

Notes: SPAD, leaf chlorophyll relative content; Fv/Fm (variable chloroplast fluorescence/maximal chloroplast fluorescence) leaf maximum photochemical efficiency. The software SPSS 19.0 was used to perform the test of significance of difference of physiological data SPAD and Fv/Fm under Duncan’s significant difference test at the *p* < 0.05 level. Different uppercase letters in the same column indicate significant differences at *p* < 0.01 level between the different species and different measurement times. All data are presented as the mean ± SE (*n* = 6), and *p* value for Fv/Fm is 0.007429.

**Table 6:** The structure and length of the chloroplast genome sequences in sugarcane hybrids and their ancestors *Saccharum officinarum* and *S. spontaneum*.

Species	*S. officinarum*		*S. spontaneum*			*S. officinarum* Hybrids					
Accession	Badila	IJ76-514	Yunnan 83-184	IJ76-287	SES234B	RB867515	Q155	SP80-3280	NCo310	RB72454	Q165
Length/bp	141,187	141,176	141,181	141,348	141,185	141,181	141,181	141,182	141,182	141,151	141,181
LSC/bp	83,065	83,042	83,047	83,226	83,063	83,047	83,047	83,048	83,048	83,017	83,047
SSC/bp	12,544	12,544	12,544	12,544	12,544	12,544	12,544	12,546	12,544	12,544	12,544
IRa/bp	22,789	22,795	22,795	22,789	22,789	22,795	22,795	22,794	22,795	22,795	22,795
IRb/bp	22,789	22,795	22,795	22,789	22,789	22,795	22,795	22,794	22,795	22,795	22,795
Accession numbers in NCBI	Obtained in this study	NC_035224.1	Obtained in this study	LS975131.1	LN849912.1	KX507245.1	NC_029221.1	NC_005878.2	NC_006084.1	LN849914.1	LN896359.1

## Supplementary Materials

Supplementary materials can be found at https://www.mdpi.com/1422-0067/20/15/3828/s1.
