# Supplementary material for: Comparative Analysis of two Sugarcane Ancestors Saccharum officinarum and S. spontaneum based on Complete Chloroplast Genome Sequences and Photosynthetic Ability in Cold Stress
Source: Int J Mol Sci. 2019 Aug 5;20(15):3828. doi: 10.3390/ijms20153828 (PMC6696253; doi:10.3390/ijms20153828)
Supplement: Supplementary file 1 [file ijms-20-03828-s001.zip › Suplementary materials_Sugarcane Cp genome and photosynthetic ability/Supplementary Figures and Table_Sugarcane cp genome and photosynthetic ability-20190707.docx]

Article

Comparative analysis of two sugarcane ancestors *Saccharum officinarum* and *S. spontaneum* based on complete chloroplast genome sequences and photosynthetic ability in cold stress

Fu Xu^1,2^, Lilian He^1^, Shiwu Gao^2^, Yachun Su^2^, Fusheng Li^1, 2^* and Liping Xu ^2^*

^1^ College of Agronomy and Biotechnology, Yunnan Agricultural University, Kunming 650201, China; [xufu1992@126.com](mailto:xufu1992@126.com) (F.X.); [helilian905@sohu.com](mailto:helilian905@sohu.com) (L.H.); [gaoshiwu2008@126.com](mailto:gaoshiwu2008@126.com) (S.G.); [syc2009mail@163.com](mailto:syc2009mail@163.com) (Y.S.); [lfs810@sina.com](mailto:lfs810@sina.com) (F.L); [xlpmail@126.com](mailto:xlpmail@126.com) (L.X.)

^2^ Key Laboratory of Sugarcane Biology and Genetic Breeding, Fujian Agriculture and Forestry University, Fuzhou 350002, China; xlpmail@126.com (L.X.)

***** Correspondence: lfs810@sina.com, xlpmail@126.com; Tel.: +86-871-6522-0502 (F.L.); +86-591-8377-2604 (L.X.)

**Supplementary Materials**


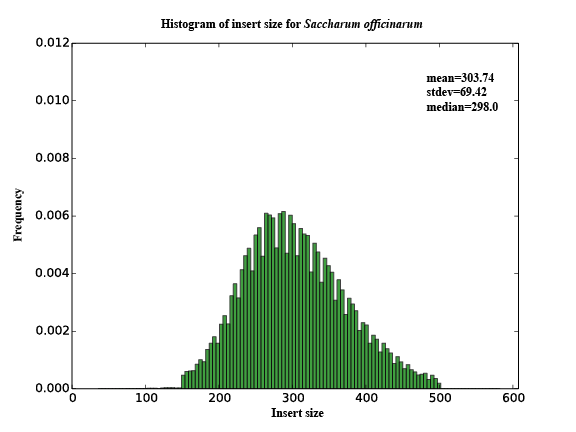


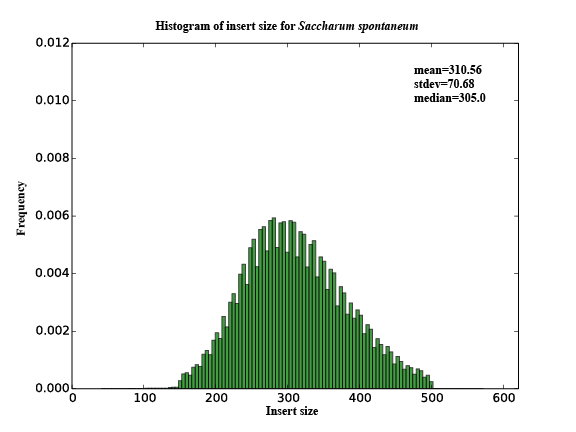


**Figure S1**. The histogram of insert size for *Saccharum officinarum* and *S. spontaneum*. The insert size is indicated in abscissa axis, and the frequency corresponding to the specific insert size is indicated in ordinate axis.


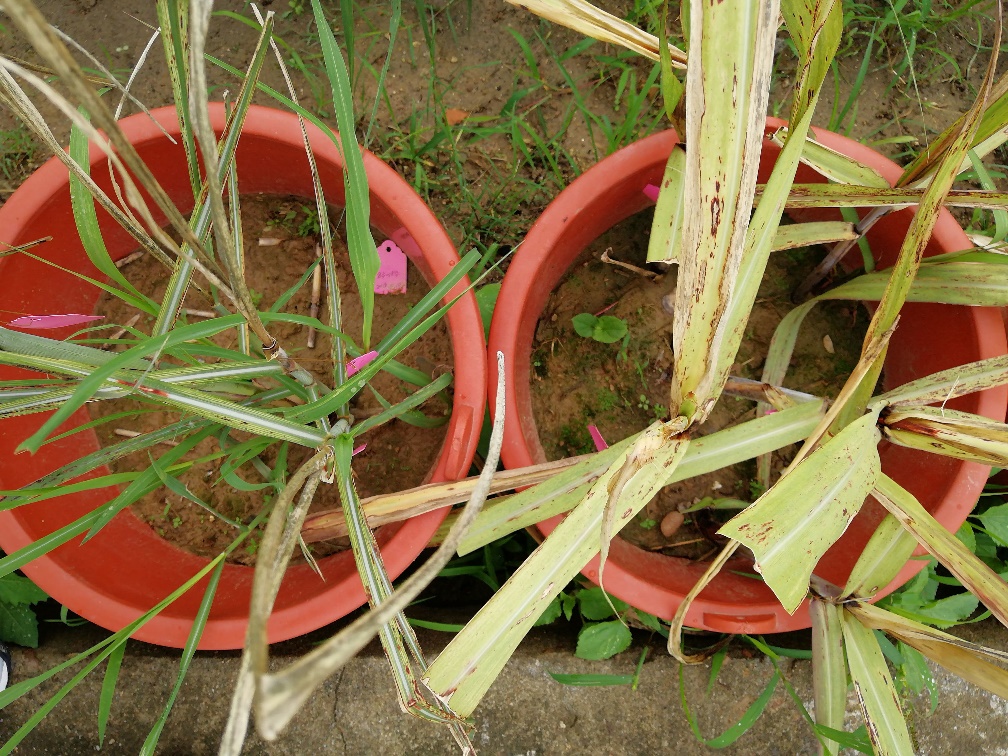


**Figure S2**. The plantlets of *Saccharum officinarum* (at right side) and *S. spontaneum* (at left side) during recovery for two weeks after removing from 4 °C incubator. Indicating several new tillers grown in wild species *S. spontaneum*, while non tiller appeared in *S. officinarum.*

**Table S1**. Detailed information of gene names, their corresponding sequences and exons/introns existed in them in *Saccharum officinarum* (See in excel Table S1.)

**Table S2**. Detailed information of gene names, their corresponding sequences and exons/introns existed in them in *Saccharum spontaneum* (See in excel Table S2.)

**Table S3**. Frequency of identified SSR motifs in chloroplast genome sequence of *Saccharum spontaneum* (Yunnan 83-184)

| Repeat types^1^ | Frequency of Repeats | | | | | | | | | | | | | Total frequency |
| --- | --- | --- | --- | --- | --- | --- | --- | --- | --- | --- | --- | --- | --- | --- |
|  | 3 | 4 | 5 | 6 | 7 | 8 | 9 | 10 | 11 | 12 | 13 | 14 | 15 |  |
| A/T | - | - | - | - | - | 63 | 29 | 16 | 7 | 3 | 1 | 3 | 1 | 123 |
| C/G | - | - | - | - | - | 2 | 2 | 1 |  |  |  |  |  | 5 |
| AG/CT | - | - | 1 |  |  |  |  |  |  |  |  |  |  | 1 |
| AT/AT | - | - | 4 |  |  |  |  |  |  |  |  |  |  | 4 |
| AAC/GTT | 9 |  |  |  |  |  |  |  |  |  |  |  |  | 9 |
| AAG/CTT | 21 | 1 |  |  |  |  |  |  |  |  |  |  |  | 22 |
| AAT/ATT | 9 |  |  |  |  |  |  |  |  |  |  |  |  | 9 |
| ACT/AGT | 1 |  |  |  |  |  |  |  |  |  |  |  |  | 1 |
| AGC/CTG | 3 |  |  |  |  |  |  |  |  |  |  |  |  | 3 |
| AGG/CCT | 1 |  |  |  |  |  |  |  |  |  |  |  |  | 1 |
| ATC/ATG | 2 |  |  |  |  |  |  |  |  |  |  |  |  | 2 |
| AAAG/CTTT | 2 |  |  |  |  |  |  |  |  |  |  |  |  | 2 |
| AAAT/ATTT | 1 |  |  |  |  |  |  |  |  |  |  |  |  | 1 |
| AACG/CGTT | 2 |  |  |  |  |  |  |  |  |  |  |  |  | 2 |
| ATC/ATG | 2 |  |  |  |  |  |  |  |  |  |  |  |  | 1 |
| AAGG/CCTT | 1 |  |  |  |  |  |  |  |  |  |  |  |  | 1 |
| AATC/ATTG | 1 |  |  |  |  |  |  |  |  |  |  |  |  | 1 |
| ACCT/AGGT | - | 1 |  |  |  |  |  |  |  |  |  |  |  | 1 |
| ATCC/ATGG | 1 |  |  |  |  |  |  |  |  |  |  |  |  | 1 |
| AATAT/ATATT | 1 |  |  |  |  |  |  |  |  |  |  |  |  | 1 |
| Total number |  |  |  |  |  |  |  |  |  |  |  |  |  | 190 |

Note: Definition of microsatellites (unit size / minimum number of repeats): (1/8) (2/5) (3/3) (4/3) (5/3) (6/3); ^1^ Frequency of classified repeat types (considering sequence complementary); SSRs present in compound formation are followings: (TATAA)3ttaat(ATA)3 (size 29 bp) at the position 21,020-21,048, (A)9t(A)8 (size 18 bp) at the position 65,491-65,508, and (T)10ctctccta(T)10 (size 28 bp) at the position 79,031-79,058.

**Table S4**. Frequency of identified SSR motifs in chloroplast genome sequence of *Saccharum officinarum* (Badila)

| Repeat types^1^ | Frequency of Repeats | | | | | | | | | | | | Total frequency |
| --- | --- | --- | --- | --- | --- | --- | --- | --- | --- | --- | --- | --- | --- |
|  | 3 | 4 | 5 | 6 | 7 | 8 | 9 | 10 | 11 | 12 | 13 | 14 |  |
| A/T | - | - | - | - | - | 61 | 30 | 15 | 7 | 3 | 2 | 2 | 120 |
| C/G | - | - | - | - | - | 3 | 2 | 1 |  |  |  |  | 6 |
| AG/CT | - | - | 1 |  |  |  |  |  |  |  |  |  | 1 |
| AT/AT | - | - | 4 |  |  |  |  |  |  |  |  |  | 4 |
| AAC/GTT | 9 |  |  |  |  |  |  |  |  |  |  |  | 9 |
| AAG/CTT | 21 | 1 |  |  |  |  |  |  |  |  |  |  | 22 |
| AAT/ATT | 9 |  |  |  |  |  |  |  |  |  |  |  | 9 |
| ACT/AGT | 1 |  |  |  |  |  |  |  |  |  |  |  | 1 |
| AGC/CTG | 3 |  |  |  |  |  |  |  |  |  |  |  | 3 |
| AGG/CCT | 1 |  |  |  |  |  |  |  |  |  |  |  | 1 |
| ATC/ATG | 2 |  |  |  |  |  |  |  |  |  |  |  | 2 |
| AAAG/CTTT | 2 |  |  |  |  |  |  |  |  |  |  |  | 2 |
| AAAT/ATTT | 1 |  |  |  |  |  |  |  |  |  |  |  | 1 |
| AACG/CGTT | 2 |  |  |  |  |  |  |  |  |  |  |  | 2 |
| AAGG/CCTT | 1 |  |  |  |  |  |  |  |  |  |  |  | 1 |
| ACCT/AGGT | - | 1 |  |  |  |  |  |  |  |  |  |  | 1 |
| ATCC/ATGG | 1 |  |  |  |  |  |  |  |  |  |  |  | 1 |
| AAAGT/ACTTT | 1 |  |  |  |  |  |  |  |  |  |  |  | 1 |
| AATAT/ATATT | 1 |  |  |  |  |  |  |  |  |  |  |  | 1 |
| Total number |  |  |  |  |  |  |  |  |  |  |  |  | 189 |

Note: Definition of microsatellites (unit size / minimum number of repeats): (1/8) (2/5) (3/3) (4/3) (5/3) (6/3); ^1^ Frequency of classified repeat types (considering sequence complementary); SSRs present in compound formation are followings: (TATAA)3ttaat(ATA)3 (size 29 bp) at the position 21,028-21,056, (A)9t(A)11 (size 21 bp) at the position 65,503-65,523, and (T)10ctctccta(T)10 (size 28 bp) at the position 79,044-79,071.

**Table S5.** SSRs identified in chloroplast genome of two sugarcane ancestors *Saccharum officinarum* and *S. spontsneum* (See in excel Table S5.)
